# Immunobiotic Bifidobacteria Strains Modulate Rotavirus Immune Response in Porcine Intestinal Epitheliocytes via Pattern Recognition Receptor Signaling

**DOI:** 10.1371/journal.pone.0152416

**Published:** 2016-03-29

**Authors:** Takamasa Ishizuka, Paulraj Kanmani, Hisakazu Kobayashi, Ayako Miyazaki, Junichi Soma, Yoshihito Suda, Hisashi Aso, Tomonori Nochi, Noriyuki Iwabuchi, Jin-zhong Xiao, Tadao Saito, Julio Villena, Haruki Kitazawa

**Affiliations:** 1 Food and Feed Immunology Group, Laboratory of Animal Products Chemistry, Graduate School of Agricultural Science, Tohoku University, Sendai, Japan; 2 Livestock Immunology Unit, International Education and Research Center for Food and Agricultural Immunology (CFAI), Graduate School of Agricultural Science, Tohoku University, Sendai, Japan; 3 Viral Diseases and Epidemiology Research Division, National Institute of Animal Health, NARO, Tsukuba, Japan; 4 Research and Development Section, Institute of Animal Health, JA Zen-noh (National Federation of Agricultural Cooperative Associations), Chiba, Japan; 5 Department of Food, Agriculture and Environment, Miyagi University, Sendai, Japan; 6 Cell Biology Laboratory, Graduate School of Agricultural Science, Tohoku University, Sendai, Japan; 7 Infection Immunology Unit, International Education and Research Center for Food Agricultural Immunology (CFAI), Graduate School of Agricultural Science, Tohoku University, Sendai, Japan; 8 Food Science and Technology Institute, Morinaga Milk Industry Co. Ltd, Zama, Kanagawa, Japan; 9 Laboratory of Immunobiotechnology, Reference Centre for Lactobacilli (CERELA-CONICET), Tucuman, Argentina; Tulane University, UNITED STATES

## Abstract

In this work, we aimed to characterize the antiviral response of an originally established porcine intestinal epithelial cell line (PIE cells) by evaluating the molecular innate immune response to rotavirus (RVs). In addition, we aimed to select immunomodulatory bacteria with antiviral capabilities. PIE cells were inoculated with RVs isolated from different host species and the infective titers and the molecular innate immune response were evaluated. In addition, the protection against RVs infection and the modulation of immune response by different lactic acid bacteria (LAB) strains was studied. The RVs strains OSU (porcine) and UK (bovine) effectively infected PIE cells. Our results also showed that RVs infection in PIE cells triggered TLR3-, RIG-I- and MDA-5-mediated immune responses with activation of IRF3 and NF-κB, induction of IFN-β and up-regulation of the interferon stimulated genes MxA and RNase L. Among the LAB strains tested, *Bifidobacterium infantis* MCC12 and *B*. *breve* MCC1274 significantly reduced RVs titers in infected PIE cells. The beneficial effects of both bifidobacteria were associated with reduction of A20 expression, and improvements of IRF-3 activation, IFN-β production, and MxA and RNase L expressions. These results indicate the value of PIE cells for studying RVs molecular innate immune response in pigs and for the selection of beneficial bacteria with antiviral capabilities.

## Introduction

Rotavirus (RVs) genome is constituted by 11-segmented double strand RNA (dsRNA) encoding structural and non-structural proteins that allow virus to effectively infect intestinal epithelial cells (IECs) [1]. RVs infect mainly the villi of the small intestine causing apical cell death and necrosis of apical villi, which results in lower digestion, primary maladsorption and acute diarrhea [2, 3]. RVs is a leading etiologic agent of viral gastroenteritis in young animals, especially in suckling and weaned piglets [4, 5]. Therefore, it is crucial to investigate immune responses to RVs infection and to obtain a clear picture of viral pathogenesis in the pig in order to develop new strategies that can be used to reduce rotaviral infections in animals.

The innate immune response is critical for limiting RVs replication and disease in the host [6]. In this regard, IECs have a crucial role in the defense against RVs through their capacity to express pattern recognition receptors (PRRs) able to sense viral molecules. Toll-like receptor (TLR)-3 is able to recognize dsRNA of RVs, leading to the activation of interferon (IFN) regulatory factors (IRFs) and nuclear factor (NF)-κB [1, 7]. Both IRFs (IRF3 and IFR7) and NF-κB are able to induce the production of INFs, especially type-I IFNs [8]. In addition, retinoic acid-inducible gene 1 (RIG-1, also known as Ddx58) and, melanoma differentiation-associated gene 5 (MDA-5, also known as lfih1 or helicard) are able to sense RVs dsRNA and trigger the complex signal cascade that induce the production of IFNs by binding with IFN-β promoter stimulator 1 (IPS-1), which is also known as mitochondrial antiviral signaling protein (MAVS) [9]. Both, IFN-α and IFN-β play important roles in controlling RVs infection since the secretion of type I IFN results in the expression of several hundred IFN stimulated gene (ISG) products with antiviral activities, both within infected cells as well as in bystander cell populations [8].

Molecular information regarding antiviral immune response against RVs in IECs has been obtained *in vitro* by using cell lines of different origins. Studies have used human colon adenocarcinoma (Caco-2) and carcinoma (HT-29) cell lines, and Madin-Darby canine kidney (MDCK) and rhesus monkey kidney (MA104) cell lines to study RVs infection or host-pathogen interactions *in vitro* (reviewed in [10]). Of interest, Caco-2 and HT-29 cells are tumorigenic lines and it was found that they possess different phenotypes compared with normal cells therefore; they would not be able to mimic exactly the behavior of IECs in response to the challenge with RVs [11]. The porcine small intestinal epithelial cell line (IPEC-J2) has been proposed as model for the study of innate immune responses to RVs. It was demonstrated that porcine RVs are able to replicate in this cell line to a high titer and induce a potent inflammatory response. Moreover, this cell line has been used for the selection and study of immunobiotic bacteria able to beneficially modulate antiviral immune response [12, 13]. However, no detailed molecular studies have been performed in RVs-infected porcine IECs.

Our research group has used an originally established porcine intestinal epithelial cell line (PIE cells) for the study of TLR3-triggered immune response in IECs and for the selection of lactic acid bacteria (LAB) strains with specific immunomodulatory properties, considering that approaches aiming to modulate pathways leading to IFNs production may provide valuable tools to increase natural viral defense mechanisms [14, 15]. We evaluated the response of PIE cells to poly(I:C) challenge and found that monocyte chemotactic protein 1 (MCP-1), interleukin (IL)-8, tumor necrosis factor (TNF)-α, IL-6 and both IFN-α and IFN-β were up-regulated in PIE cells after stimulation indicating that PIE cells are a good tool to study *in vitro* the immune responses triggered by TLR3 on IECs. We also showed that our *in vitro* system could be used for the selection of immunobiotic LAB strains with anti-viral and immune enhancing activities [14, 15]. Moreover, PIE cells have allowed us to perform detailed molecular studies to gain insight into the mechanisms involved in immunobiotic effects by evaluating PRRs expression, activation of signaling pathways and modulation of PRRs-negative regulators [16].

In this work we aimed to evaluate whether PIE cells are a useful tool for studying the molecular innate immune response to RVs infection and for the selection of immunomodulatory bacteria with antiviral capabilities.

## Materials and Methods

### Epithelial cell lines

The porcine intestinal epithelial cell line (PIE cells) is a non-transformed intestinal cell line, which is originally established in our laboratory from intestinal epithelia of unsuckled neonatal swine [17]. PIE cells are characterized by its ability to build a monolayer with a cobblestone and epithelial-like morphology and close contacts between cells. These cells grow rapidly and are well acclimated to culture growth conditions without any transformation or immortalization [17, 18]. Briefly, PIE cells were grown on type I collagen coated dishes using DMEM (Gibco, Japan) supplemented with 10% fetal calf serum (FCS, Sigma). PIE cells were incubated at 37°C and 5% CO_2_. Passages were done by treating monolayers with sucrose/EDTA for 4 min and detaching the cells with the addition of 0.04% trypsin. The rhesus monkey kidney cell line MA104 was cultured in minimal essential medium (MEM) supplemented with 10% heat-inactivated fetal bovine serum (FBS) at 37°C in a humidified atmosphere of 5% CO_2_.

### Analysis of microvilli

Development of microvilli on the surface of PIE cells was examined using scanning electron microscopy (SEM). PIE cells were seeded on 12 well type I collagen coated cover glass (IWAKI, Tokyo, Japan) at 9 × 10^5^ cells/well. After 3 and 10 days of incubation, cells were washed once with 0.1 M phosphate buffer (pH 7.4) and then fixed with 2.5% glutaraldehyde in 0.1 M phosphate buffer for 1 hour. Following three rounds of washing with 0.1 M phosphate buffer, the cells were dehydrated by passage through graded dilutions of ethanol and substituted with t-butylalcohol. Cells were freeze-dried, coated with platinum-palladium and observed under the SEM microscope (S4200, Hitachi, Tokyo, Japan).

### Lactic acid bacterial strains

*Bifidobacterium longum* MCC1, *B*. *infantis* MCC12, *B*. *breve* MCC167, MCC1274, *B*. *pseudolongum* MCC92, *Lactobacillus paracasei* MCC1375, *L*. *gasseri* MCC587, and *Lactococcus lactis* sub sp. *lactis* MCC866 were used in this work. These strains were provided by Morinaga Milk Industry Co., Ltd. (Zama, Japan). Bifidobacterium strains were incubated in Man-Rogosa-Sharpe (MRS) broth (Difco, Detroit, MI, USA) supplemented with 0.05% (w/v) cysteine (Sigma, Tokyo, Japan) at 37°C for 16 hours under anaerobic conditions (AnaeroGen; Oxoid, Basingstoke, UK). Lactobacillus strains were grown in MRS broth at 37°C for 16 hours. Cultures were then centrifuged at 1900 g for 15 minutes, and the bacteria were washed with phosphate-buffered saline (PBS), and resuspended in DMEM cell culture medium at the appropriate concentrations [19].

### Rotavirus strains

Four RVs strains isolated from different host species including human (Wa), murine (EW), bovine (UK) and porcine (OSU) were used in this study. Serotypic characterization of RVs derived from asymptomatic human neonatal infections [20]. Briefly, RVs strains were treated with 10 μg/ml trypsin (Sigma, Type I) at 37°C for 30 minutes and inoculated onto confluent MA104 cells. After an hour of absorption, the inoculum was removed and the cells were incubated with serum-free MEM containing 1 μg/ml trypsin at 37°C. When the cytopathic effect reached more than 80%, the culture supernatant was harvested by three rounds of freezing and thawing process. The virus stock was stored at -80°C for further experiments.

### Rotavirus infectivity in PIE cells

PIE cells were seeded at a density of 5.0 × 10^3^ cells/well in 96 well microplate coated with type I collagen (SUMILON, Tokyo, Japan). Cells were cultured for 3 or 10 days and then inoculated with trypsin-treated RVs strains diluted serially 10-folds or adjusted to multiplicity of infection (MOI) in serum-free DMEM containing 1 μg/ml of trypsin. At hour 16 post-inoculation, the cells were fixed after removal of the inoculums and the infected virus titer were analyzed by immunofluorescence staining.

### Immunofluorescence staining for detection of cells infected with rotavirus

RVs challenged-cells were fixed with 80% acetone at 4°C for 15 minutes. Then, cells were washed twice with PBS, and subsequently incubated with a guinea pig anti-RVs Wa strain polyclonal antibody (1:750 in PBS, 50μl/well) for 30 minutes at 37°C. Following three washes with PBS, cells were incubated at 37°C for 30 minutes with Fluorescein isothiocyanate (FITC) conjugated anti-guinea pig IgG (H+L) antibody (Rockland antibodies and assays, Limerick, PA, 1:400 in PBS, 50 μl/well). Infected cells were examined and photographed under an immunofluorescence microscope (Confocal laser microscope, MRC-1024, Bio-Rad, Richmond, CA) after three rounds of washing with PBS and mounted with 30% glycerol prepared in PBS.

### Expression of anti-viral and inflammatory factors in rotavirus infected PIE cells

PIE cells were plated at density of 3.0 × 10^4^ cells in 12 well type I collagen coated plates (SUMILON, Tokyo, Japan) and cultured at 37°C, 5% CO_2_. Ten days later, cells were inoculated with trypsin-activated OSU or UK RVs strains at MOI 1. At 0, 3, 6, and 12 hours post-inoculation, cells were washed twice with PBS and treated with 500 μl of TRIzol reagent (Life technologies, CA) to collect total RNA according to the manufacturer’s instruction. After total RNA extraction, cDNAs were synthesized by using a Quantitect reverse transcription (RT) kit (Qiagen, Tokyo, Japan). The expression of CXCL10, IL-6, IL-8, MCP-1, IFN-β, MxA, RNaseL, RIG-I, and TLR3 were quantified by performing real-time quantitative PCR using the primer sets listed in S1 Table, and platinum SYBR green qPCR supermix uracil-DNA glycosylase with 6-carboxyl-X-rhodamine (Invitrogen) on a 7300 real-time PCR system (Applied Biosystems, Warrington, United Kingdom). The total volume of reaction mixture was 10 μl, which contained 2.5 μl of cDNA and 7.5 μl of master mix included with SYBR green, sense, and anti-sense primers. Amplification was performed at 50°C for 5 minutes; followed by 95°C for 5 minutes; 40 cycles at 95°C for 15 s, 60°C for 30 s and 72°C for 30 s. In these experiments, β-actin was used as an internal control to normalize cDNA levels for differences in total cDNA levels in the samples.

### Effect of lactic acid bacteria on PIE cells response to poly(I:C) challenge

In order to evaluate the modulatory effect of LAB, PIE cells were plated (3.0 × 10^4^ cells/well) in 12 well type I collagen coated plates (SUMILON, Toky, Japan) and incubated at 37°C, 5% CO_2_ for 3 days. Then, the cultured cells were incubated with different LAB strains (*B*. *longum* MCC1, *B*. *infantis* MCC12, *B*. *breve* MCC167, MCC1274, *B*. *pseudolongum* MCC92, *L*. *paracasei* MCC1375, *L*. *gasseri* MCC587 or *L*. *lactis* sub sp. *lactis* MCC866) at density of 5 × 10^8^ cells/ml for 48 hours. After washing with fresh DMEM medium, the cells were stimulated with poly(I:C) (20 μg/ml) for 12 hours. After stimulation, cells were vigorously washed and harvested for total RNA isolation. The mRNA expression level of INF-β was quantified by qRT-PCR as explained before. In addition, SEM analysis was performed to evaluate the attachment of LAB on the surface of PIE cells.

### Effect of lactic acid bacteria on PIE cells response to rotavirus challenge

PIE cells were plated at 3.0 × 10^4^ cells/well in 12 well type I collagen coated plates (SUMILON, Toky, Japan) and incubated at 37°C, 5% CO_2_. After 8 days of culturing, cells were pre-stimulated with *B*. *infantis* MCC12 or *B*. *breve* MCC1274 (5 × 10^8^ cells/ml) for 48 hours. Then, cells were washed three times with DMEM medium to eliminate the bacteria and subsequently inoculated with trypsin-activated OSU or UK RVs at MOI 1. At 6 and 12 hours post-inoculation, the cells were washed and harvested for total RNA isolation. The expression of CXCL10, IL-6, IL-8, MCP-1, IFN-β, MxA, RNaseL, RIG-I, and TLR3 were quantified by qRT-PCR as described above.

### Western blot analysis

PIE cells were stimulated with *B*. *infantis* MCC12 or *B*. *breve* MCC1274 (5 × 10^8^ cells/ml) for 48 hours. Then, the cells were washed three times with DMEM medium to eliminate the bacteria and subsequently inoculated with trypsin-activated OSU and UK RVs at MOI 1. At 0, 10, 30, 60, and 120 minutes post-inoculation, the cells were washed and resuspended with 200 μl of CellLytic M cell lysis reagent (Sigma-Aldrich, St. Louis, MO, USA), containing protease and inhibitors of phosphates (Complete mini, PhosSTOP, Roche, Mannheim, Germany). Cells were transferred to 1.5 ml of Eppendorf tubes and kept at 95°C for 5 minutes in water bath. Concentration of protein was estimated using BCA assay kit (Pierce, Rockford, IL). The lysed samples (8 μg/sample) were loaded on 10% SDS-polyacrylamide gels and separated proteins were transferred electrophoretically to a nitrocellulose membrane. Phosphorylation of tumor necrosis factor (TNF)-receptor associated factor 3 (TRAF3), and interferon regulatory factor (IRF3) were evaluated using Phospho-IRF3 (Ser396) (4D4G, Cat. #4947) rabbit antibody and TRAF3 antibody (Cat. #4729) from Cell Signaling Technology (Beverly, MA, USA) at 1000 times dilution of their original antibodies overnight at room temperature. The membranes were then stripped with Ten Minute Western Blot Re-Probe Kit (#JZ-008, Jacksun Easy Biotech, Inc., New York, USA) for the detection of each total protein using IRF-3 (D83B9, Cat. #4302) antibody and β-actin (13E5, Cat. #4970) rabbit antibody from Cell Signaling Technology. Anti-rabbit IgG, AP-linked antibody (Cat. #7054) was used as secondary antibody. The optical protein bands were detected by ECF substrate (GE Healthcare Japan Co., Tokyo, Japan) and estimated from the peak area of densitogram by using Image J software (National Institute of Health, Bethesda, MD, USA).

### Statistical analysis

For statistical analysis ANOVA procedure of SAS program, version 9.1 was used. Each relative gene expression was logarithmically transformed to logarithm, and assumed as normalized data. One-way analysis of variance was performed for each time point after infection, and then the Tukey-Kramer multi comparison was used. Comparison between reduction ratios induced by bacteria stimulation was carried out by one-way analysis of variance for the different virus. Significant differences were marked as *(p<0.05), **(p<0.01), or ***(p<0.001), respectively. For multiple comparisons between mean values, p<0.05 was considered significant and indicated with different superscript letters.

## Results

### Analysis of rotavirus infectivity in PIE cells

**Fig 1:**
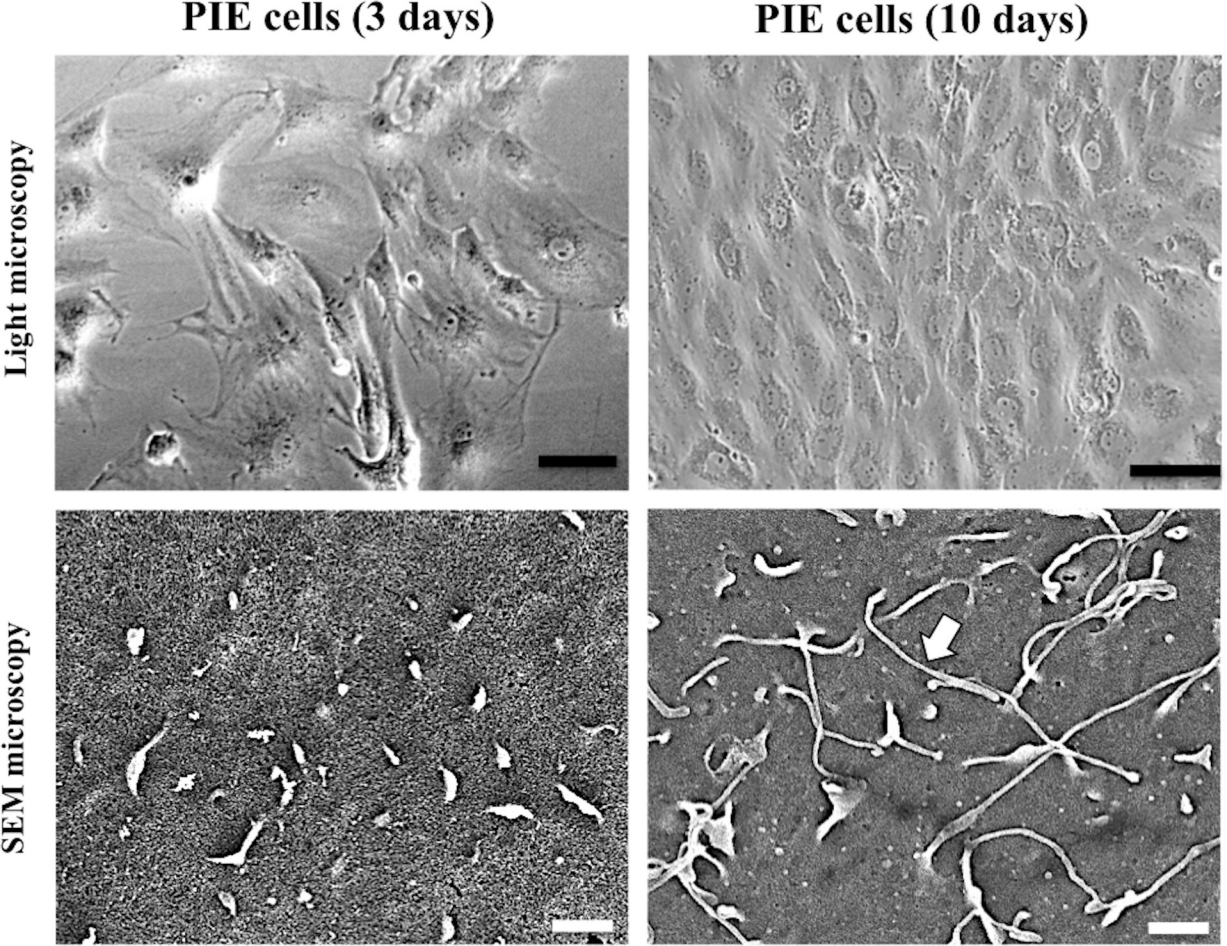
Microscopic analysis of porcine intestinal epithelial cell line (PIE cells). PIE cells were grown in DMEM medium for 3 and 10 days. The cell growth and the development of microvilli on the surface of PIE cells were analyzed by light and scanning electron microscopy (SEM) microscopies. Arrows indicate the development of microvilli. Scale bars indicate 10 μm (black) and 1 μm (white).

Considering that RVs initiate infection of IECs in microvilli, we first evaluated the development of those cellular structures on PIE cells surfaces under light and SEM microscopes after 3 and 10 days of culturing. As shown in Fig 1, PIE cells developed microvilli on their surface on day 3, and microvilli number and length were increased by extending the culturing time to 10 days.

**Fig 2:**
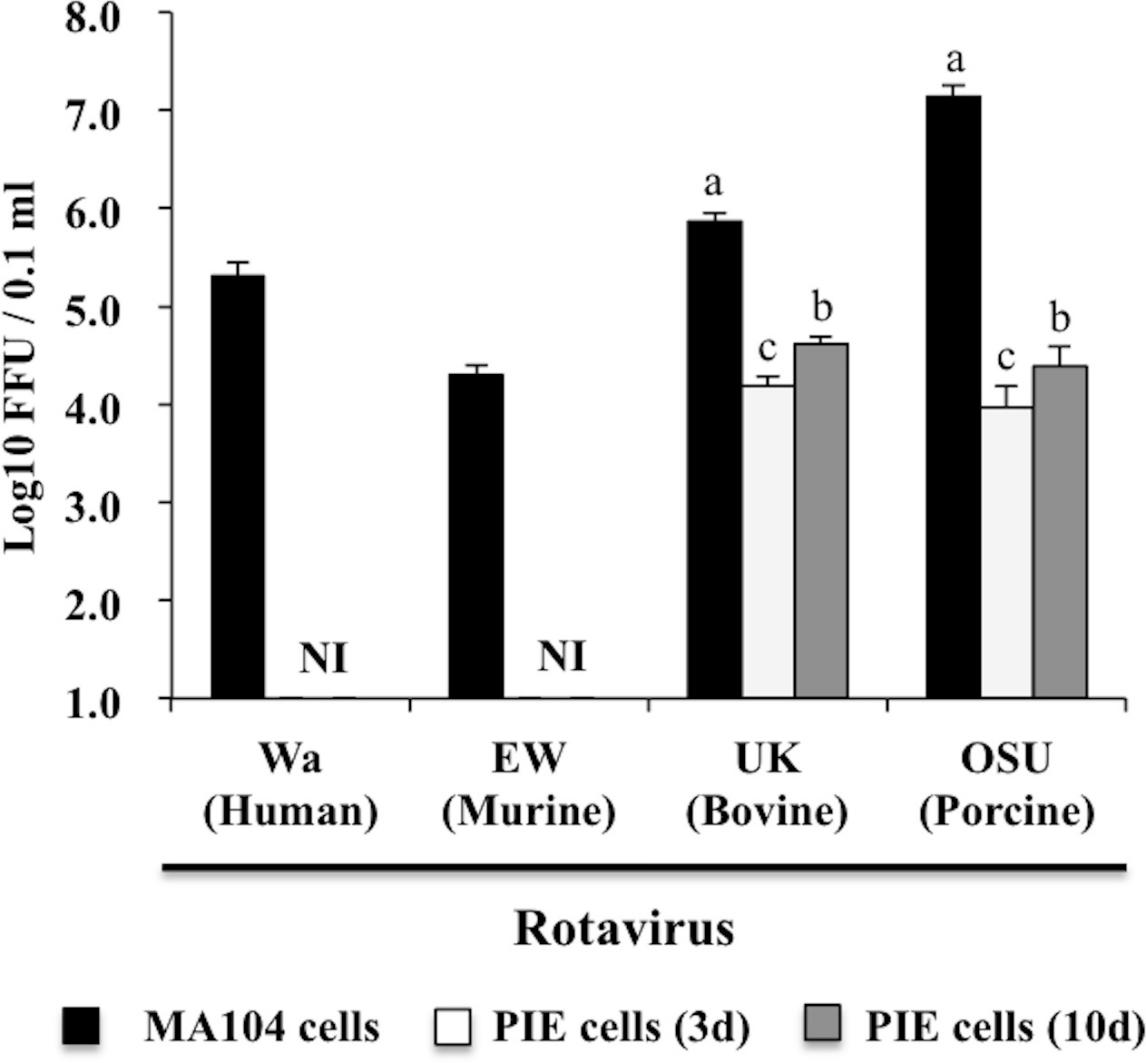
Infectivity of rotaviruses in porcine intestinal epithelial cell line (PIE cells). PIE cells cultured for 3 or 10 days. MA104 cells were used for comparison. PIE and MA104 cells were inoculated with rotaviruses from different host species including: human Wa, murine EW, bovine UK, and porcine OSU rotaviruses. Numbers of rotavirus antigen positive cells were counted by immunofluorescence assay after 16 hours post-inoculation. The virus titer was expressed as Log_10_ focus forming units (FFU)/0.1 ml. NI: no infectivity of rotaviruses. The results represent data from three independent experiments. ^a, b, c^ In the comparison within means of the same virus, the difference among means with different superscripts was significant at 5% level.

**Fig 3:**
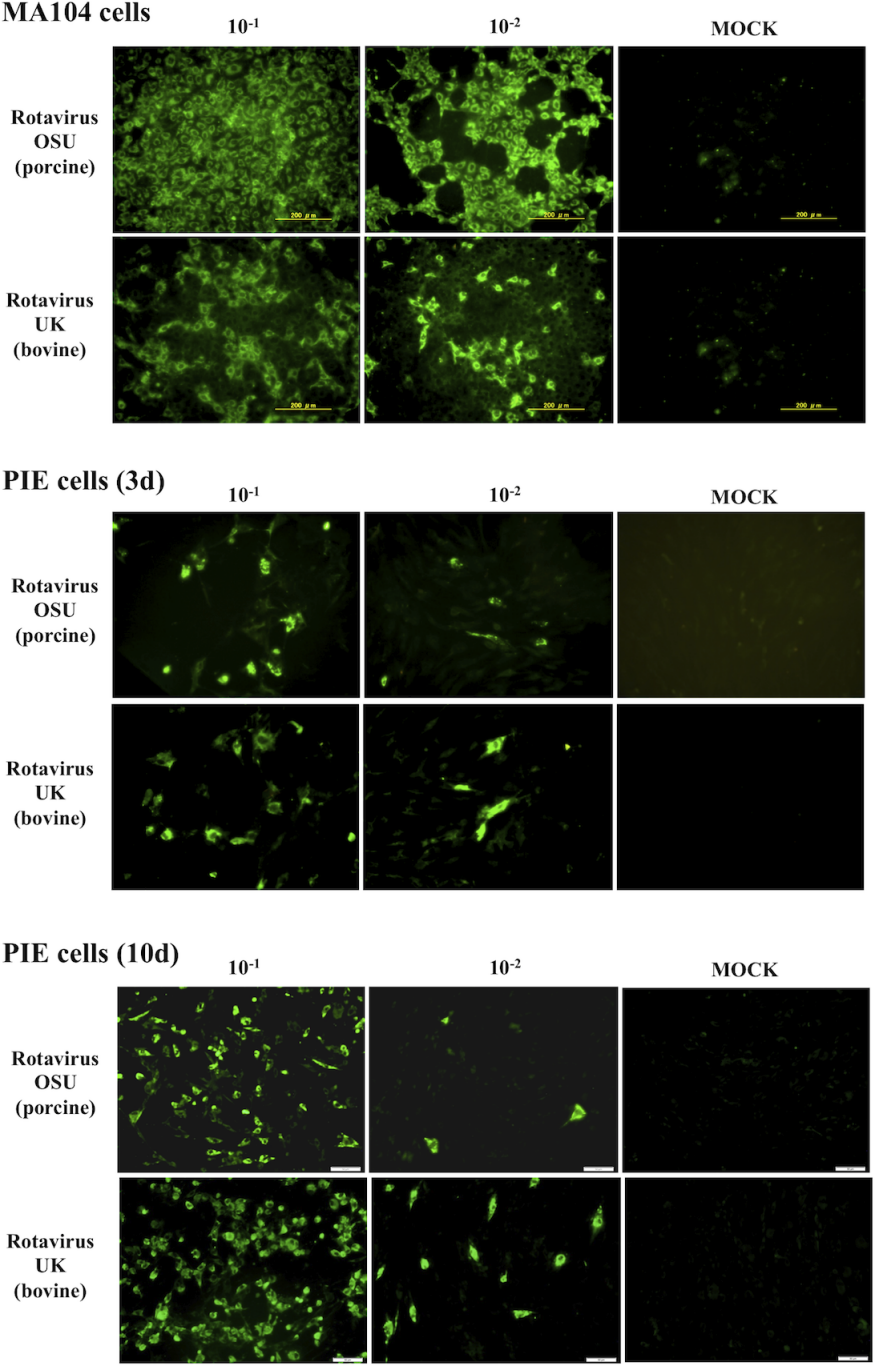
Infectivity of rotaviruses in porcine intestinal epithelial cell line (PIE cells). PIE cells cultured for 3 or 10 days. MA104 cells were used for comparison. PIE and MA104 cells were inoculated with porcine OSU or bovine UK rotaviruses and evaluated by immunofluorescence assay. The cells with specific green-fluorescence in the cytoplasm were photographed by confocal laser microscopy after labeling with fluorescence antibody.

In order to analyze RVs infectivity in PIE cells, they were cultured for 3 or 10 days, and RVs of different host origins including human (Wa), murine (EW), bovine (UK), or porcine (OSU) strains were inoculated. Experiments using MA104 cells were performed in parallel (Figs 2 and 3). The virus titers for OSU and UK RVs in PIE cells were as high as 4.4 and 4.6 log_10_ FFU/0.1 ml respectively. However, these titers were lower when compared to those obtained in MA104 cells with values of 7.1 and 5.8 log10 FFU/0.1 ml for OSU and UK RVs. Similar to infected MA104 cells, RVs antigens were observed in the cytoplasm of infected PIE cells inoculated with OSU and UK strains but not in those inoculated with Wa or EW strains (Fig 3). In addition, there were visible differences in the infectivity of 3 and 10 days cultured PIE cells. Therefore, we used 10 days cultured PIE cells for further experiments.

### Study of antiviral immune response in PIE cells

**Fig 4:**
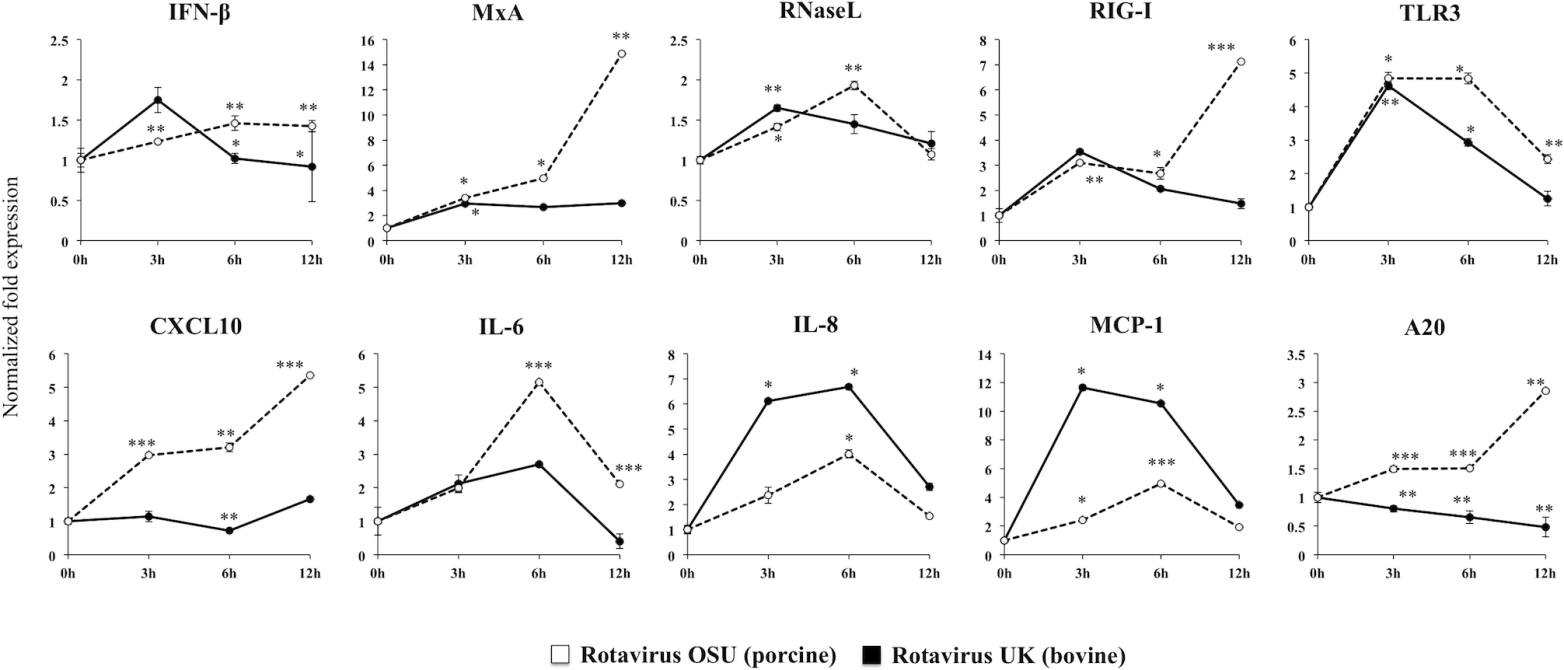
Innate immune response of porcine intestinal epithelial cell line (PIE cells) after infection with rotaviruses. PIE cells were cultured in DMEM media for 10 days, and then infected with OSU or UK rotaviruses. After 0, 3, 6, and 12 hours post-infection the expression level of IFN-β, MxA, RNaseL, RIG-I, TLR3, and cytokines (CXCL10, IL-6, IL-8, and MCP-1) were quantified. The results represent data from three independent experiments and are expressed as mean ± S.D. Asterisks indicate significant differences: *p* <0.05 (*), *p* <0.01 (**), and *p* < 0.001(***).

The expression of IFN-β, myxovirus resistance protein (MxA), 2’, 5’ oligoadinylated dependent endoribonuclease L (RNaseL), RIG-1, TLR3, cytokines (CXCL10, IL-6, IL-8, MCP-1) and the TLR negative regulator A20 were evaluated in PIE cells at 0, 3, 6, 12 hours post inoculation of OSU or UK RVs (Fig 4). Infection with both OSU and UK RVs increased the expression of IFN-β in PIE cells as well as the expression of the inflammatory cytokines and chemokines CXCL10, IL-6, IL-8 and, MCP-1. Up-regulation of IFN-β expression was more sustained in PIE cells infected with OSU strain when compared with cells challenged with UK RVs (Fig 4). Expression of CXCL10 and IL-6 was higher in OSU-infected PIE cells than in those challenged with UK RVs. On the contrary, UK-infected PIE cells showed higher levels of IL-8 and MCP-1 than OSU-infected cells (Fig 4).

Challenge with both RVs strains also increased the expression of TLR3, RIG-I, MxA and RNaseL, however the values were significantly higher in OSU-infected PIE cells than in those challenged with UK RVs (Fig 4). A20 is a zinc finger protein that inhibits activation of NF-κB via inflammatory cytokine receptors and TLRs. Therefore, A20 plays an essential role in the termination of NF-κB signaling in response to TNF-α and microbial products. Our experiments showed that OSU RVs was able to significantly increase the expression of A20 in PIE cells while UK RVs induced a down-regulation of this negative regulator (Fig 4).

### Selection of immunobiotics with antiviral effects in PIE cells

**Fig 5:**
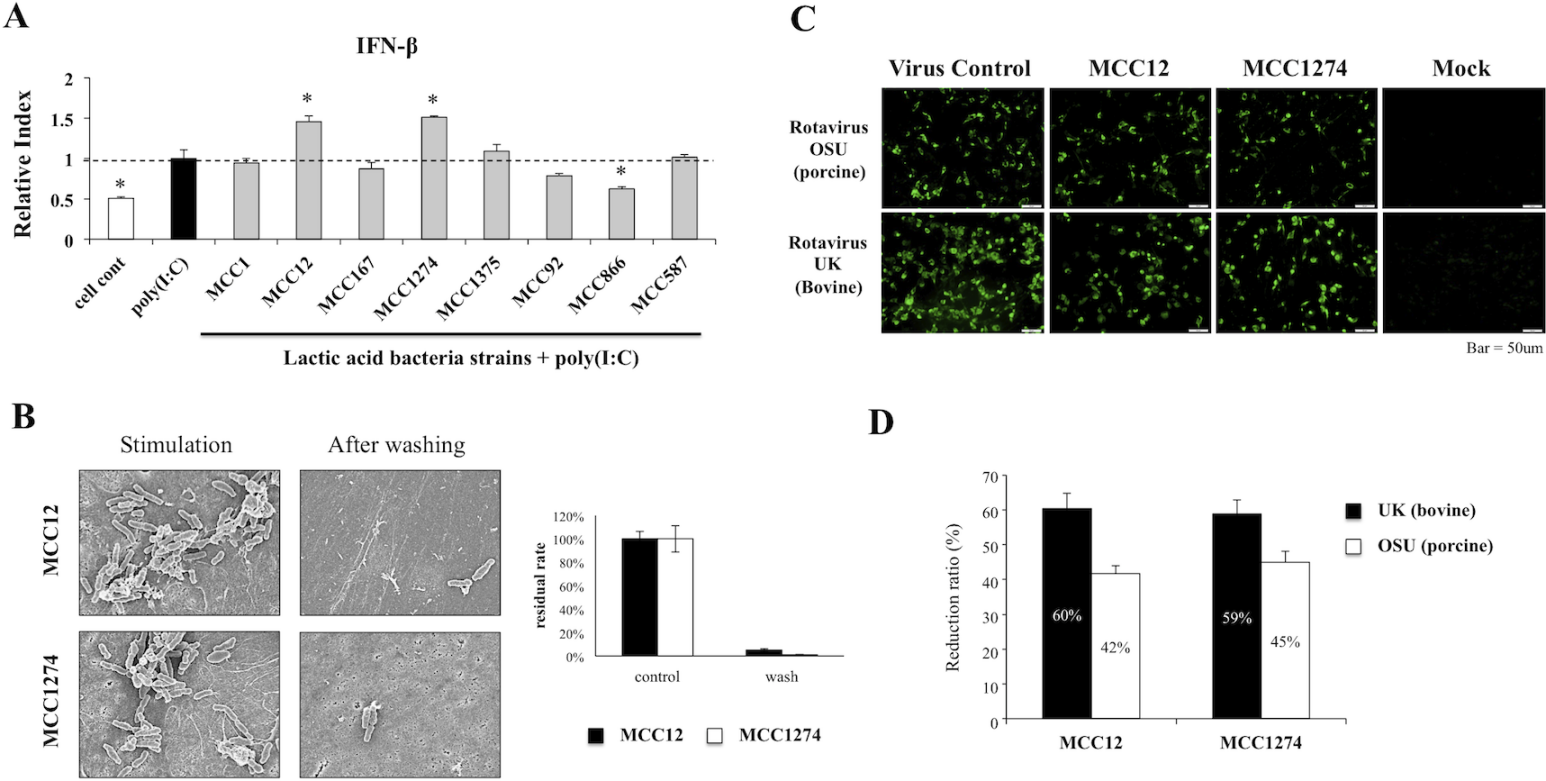
Antiviral activities of lactic acid bacteria in porcine intestinal epithelial cell line (PIE cells). (A) PIE cells were pre-stimulated with different lactic acid bacteria strains for 48 hours: *Bifidobacterium longum* MCC1, *B*. *infantis* MCC12, *B*. *breve* MCC167, MCC1274, *B*. *pseudolongum* MCC92, *Lactobacillus paracasei* MCC1375, *L*. *gasseri* MCC587, *Lactococcus lactis* sub sp. *lactis* MCC866; and then stimulated with Poly(I:C) for 12 hours. The expression level of IFN-β was quantified by RT-PCR. The results represent data from three independent experiments and are expressed as mean ± S.D. Asterisks indicate significant differences: *p* <0.05 (*). (B) Scanning electron microscopy (SEM) analysis to evaluate the attachment of *B*. *infantis* MCC12 and *B*. *breve* MCC1274 to the surface of PIE cells. (C) Effect of *B*. *infantis* MCC12 and *B*. *breve* MCC1274 on rotavirus infectivity evaluated by immunofluorescence after 12 hours of challenge. (D) The protective ability of *B*. *infantis* MCC12 and *B*. *breve* MCC1274 was examined by comparing them with untreated virus control, and calculating the reduction radio.

We previously demonstrated that the challenge of PIE cells with the TLR3/RIG-I agonist poly(I:C) could be effectively used for the screening and selection of immunobiotic strains with antiviral activity [14, 15]. Then, different LAB strains were evaluated in this work considering their capacities to improve the expression of INF-β by PIE cells in response to poly(I:C) stimulation (Fig 5A). The bacteria *B*. *longum* MCC1, *B*. *breve* MCC167, *B*. *pseudolongum* MCC92, *L*. *paracasei* MCC1375, and *L*. *gasseri* MCC587 did not induce any significant changes in the expression of INF-β compared with controls. The level of INF-β was significantly down regulated when cells were stimulated with *L*. *lactis* sub sp. *lactis* MCC866. On the contrary, *B*. *infantis* MCC12 and *B*. *breve* MCC1274 significantly increased the mRNA levels of INF-β compared with control groups (Fig 5A). Therefore, we selected *B*. *infantis* MCC12 and *B*. *breve* MCC1274 for further experiments.First, SEM was performed to analyze attachment of bifidobacteria on PIE cells. This study showed the presence of *B*. *infantis* MCC12 and *B*. *breve* MCC1274 strains on the surface of PIE cells after one hour of stimulation (Fig 5B). However, the number of bifidobacteria found on PIE cells was significantly reduced after washing with PBS, which indicates the removal of bacterial cells (Fig 5B).

We next investigated whether *B*. *infantis* MCC12 and *B*. *breve* MCC1274 were able to reduce infectivity of both OSU and UK RVs in PIE cells (Fig 5C and 5D). Pretreatment of PIE cells with MCC12 or MCC1274 prior to RVs inoculation reduced the infectivity of the viruses to around 45% in OSU and 60% in UK. These results indicate that both bifidobacteria strains have the capacity to reduce the susceptibility of PIE cells to porcine and bovine RVs.

### Study of the immunomodulatory activity of Bifidobacteria in PIE cells

**Fig 6:**
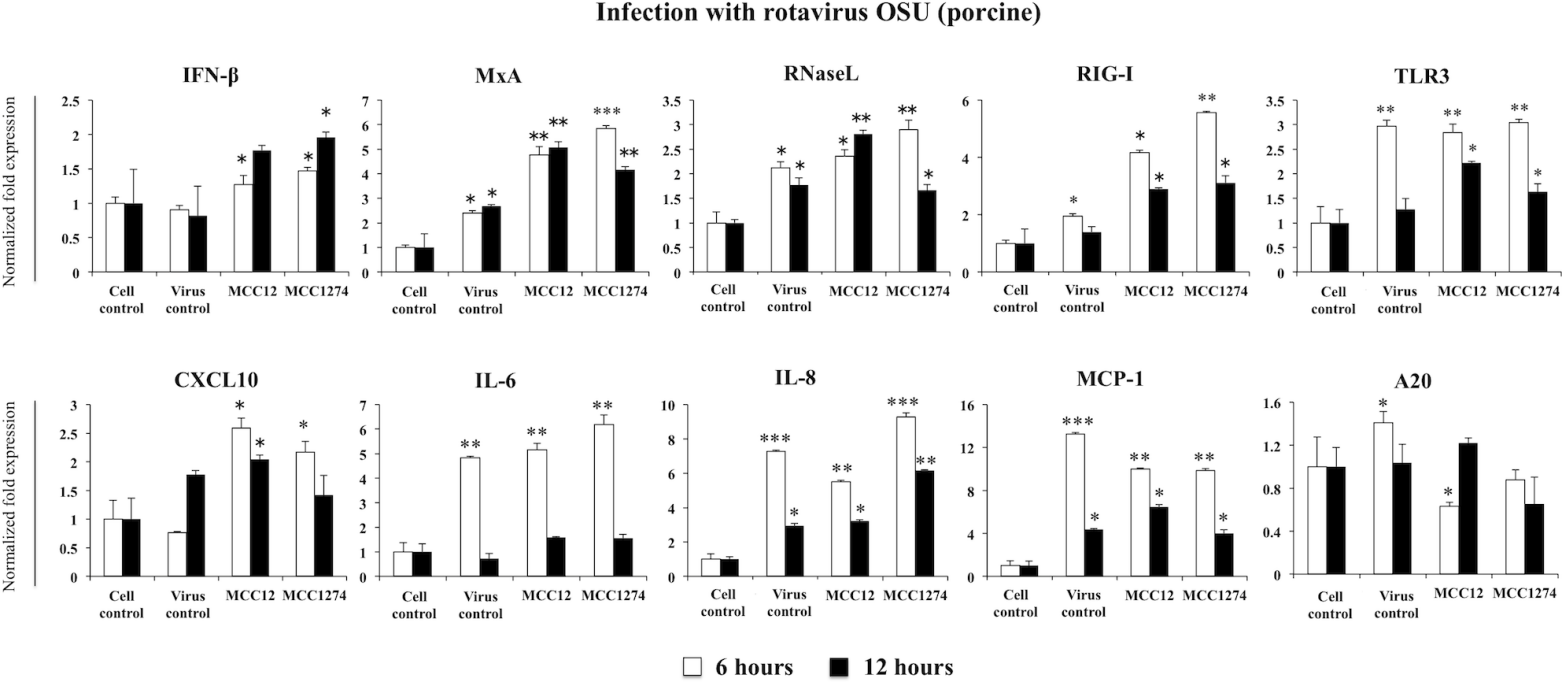
Effect of immunobiotic bifidobacteria in antiviral immune response of porcine intestinal epithelial cell line (PIE cells). PIE cells were pre-treated with *B*. *infantis* MCC12 or *B*. *breve* MCC1274 for 48 hours, and then challenged with rotavirus OSU. The expression level of IFN-β, MxA, RNaseL, RIG-I, TLR3, A20, and cytokines (CXCL10, IL-6, IL-8, MCP-1) were quantified by RT-PCR after 6 and 12 hours. The results represent data from three independent experiments and are expressed as mean ± S.D. Asterisks indicate significant differences: *p* <0.05 (*), *p* <0.01 (**), and *p* < 0.001(***).

We next aimed to study in detail the effect of selected bifidobacteria strains in the molecular innate immune response of PIE cells after RVs challenge. As shown in Fig 6, both *B*. *infantis* MCC12, and *B*. *breve* MCC1274 were able to increase the production of IFN-β in PIE cells in response to OSU RVs infection, which confirm the results obtained with poly(I:C) stimulation (Fig 5A). In addition, both bifidobacteria increased the expression of CXCL10 and IL-6 being the MCC12 strain more efficient to induce this effect. The expression of MCP-1 was significantly down-regulated in bifidobacteria-treated PIE cells when compared to controls (Fig 6). The PRRs TLR3 and RIG-I were also significantly up-regulated in bifidobacteria-treated PIE cells when compared to controls being the MCC1274 strain more efficient to increase RIG-I expression. In addition, both bifidobacteria were equally effective to improve the levels of MxA and RNaseL when compared to controls (Fig 6). Interestingly, immunobiotic treatments reduced the expression of the negative regulator A20, being the MCC12 strain more efficient to induce this effect.

**Fig 7:**
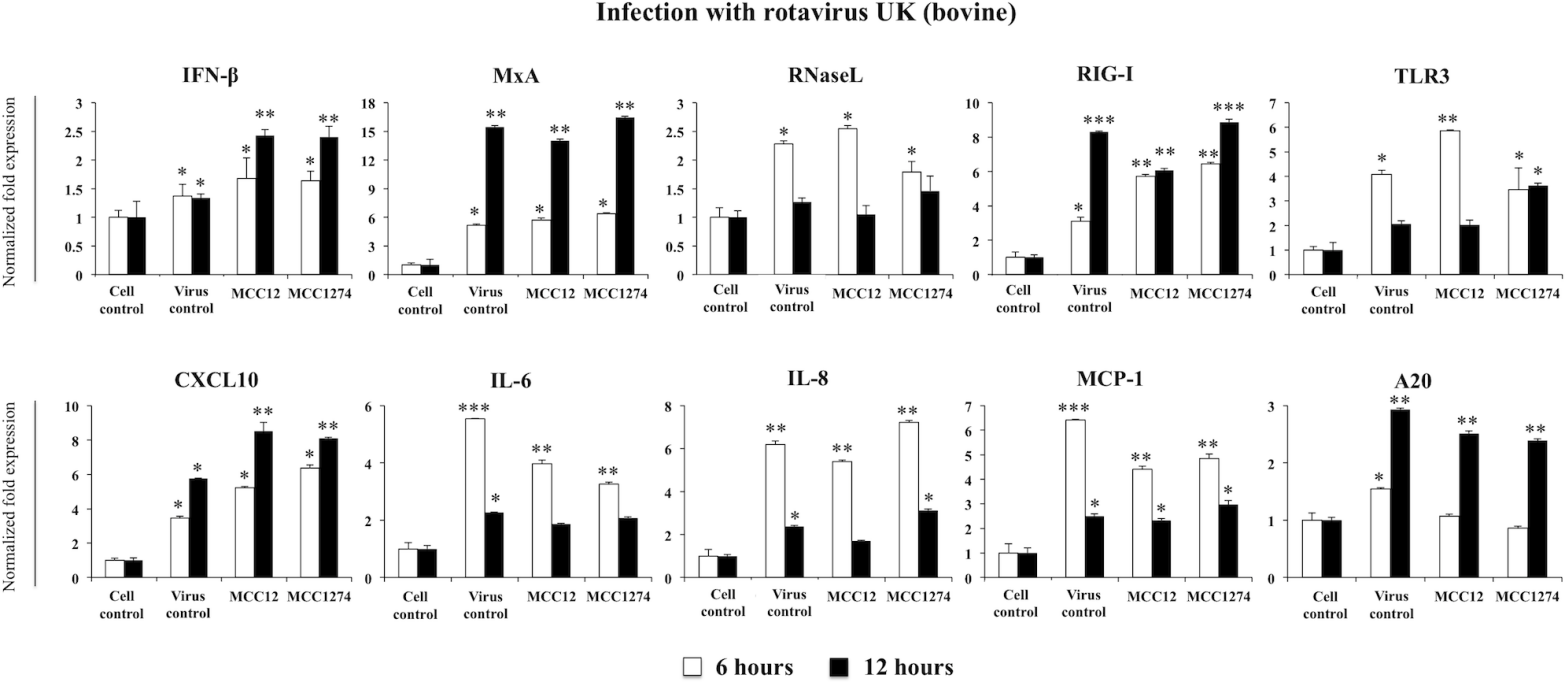
Effect of immunobiotic bifidobacteria in antiviral immune response of porcine intestinal epithelial cell line (PIE cells). PIE cells were pre-treated with *B*. *infantis* MCC12 or *B*. *breve* MCC1274 for 48 hours, and then challenged with rotavirus UK. The expression level of IFN-β, MxA, RNaseL, RIG-I, TLR3, A20, and cytokines (CXCL10, IL-6, IL-8, MCP-1) were quantified by RT-PCR after 6 and 12 hours. The results represent data from three independent experiments and are expressed as mean ± S.D. Asterisks indicate significant differences: *p* <0.05 (*), *p* <0.01 (**), and *p* < 0.001(***).

When we study the effect of *B*. *infantis* MCC12 and *B*. *breve* MCC1274 on PIE cells challenged with UK RVs, we found moderate effects when compared to those observed in OSU-infected cells. Both MCC12 and MCC1274 increased the expression of IFN-β and CXCL10 and reduced the levels of MCP-1 and IL-6 (Fig 7). No modifications were observed in the expression of RIG-I, MxA and RNaseL in bifidobacteria-treated cells when compared to controls. TLR3 was also significantly up-regulated in bifidobacteria-treated PIE cells (Fig 7). Again, as observed in OSU RVs infection experiments, immunobiotic treatments reduced the expression of A20 in UK-infected PIE cells (Fig 7).

**Fig 8:**
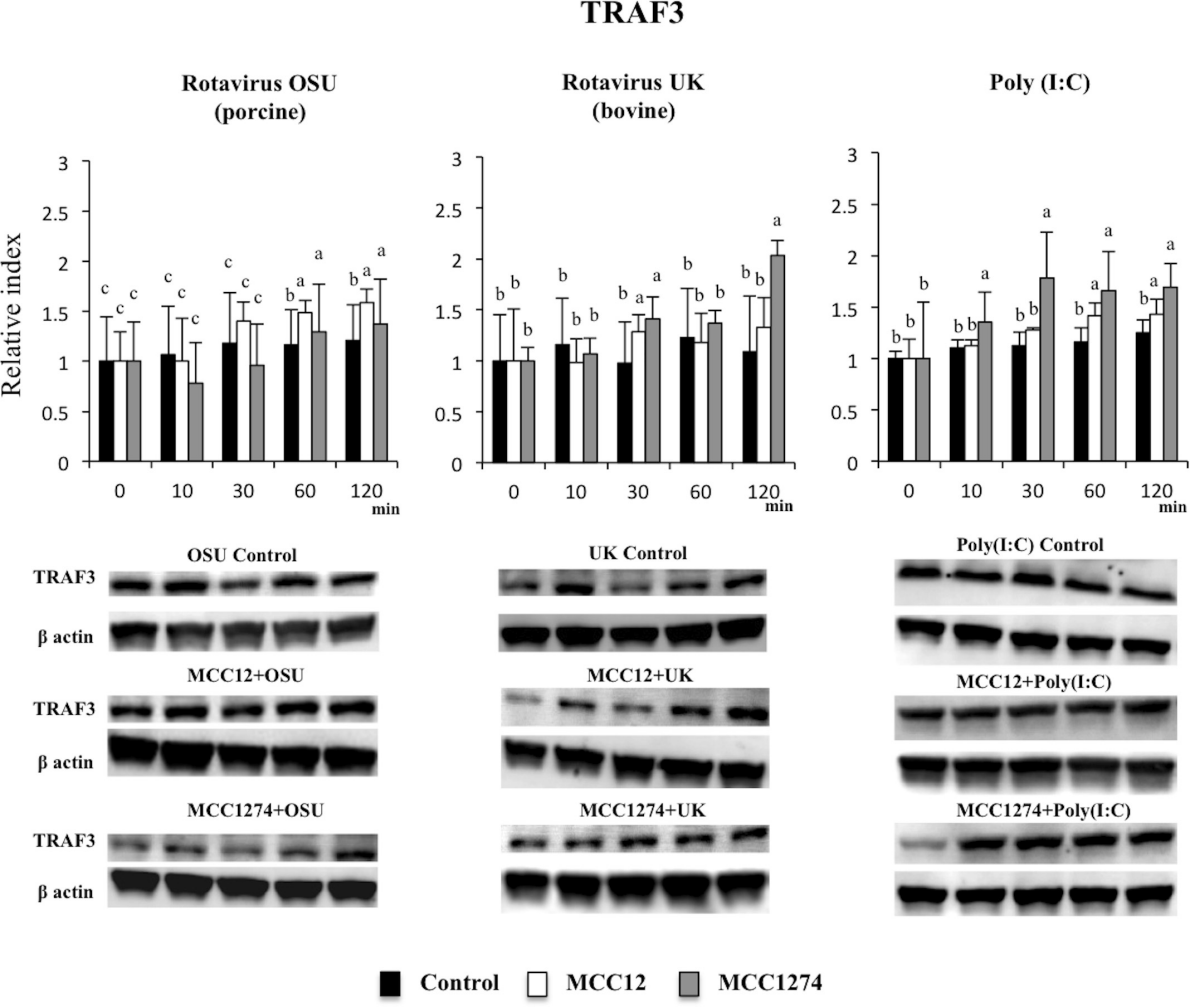
Effect of immunobiotic bifidobacteria on TRAF3 activation in porcine intestinal epithelial cell line (PIE cells). PIE cells were pre-treated with *B*. *infantis* MCC12 or *B*. *breve* MCC1274, and then challenged with rotaviruses OSU or UK; or poly(I:C). Total proteins were extracted from lysed cells and separated by SDS-PAGE, and then western-blot was performed to analyze phosphorylation of TRAF3 after 10, 30, 60 and 120 minutes. The TRAF3 specific bands were normalized to that corresponding to β-actin. Intensities of proteins bands were calculated from peak area of densitogram by using image software. The results represent data from three independent experiments and are expressed as relative index vs 0 min control with mean ± S.D. ^a, b, c^ Different superscripts letters indicate significant difference (p<0.05) among stimulants at the same time point.

**Fig 9:**
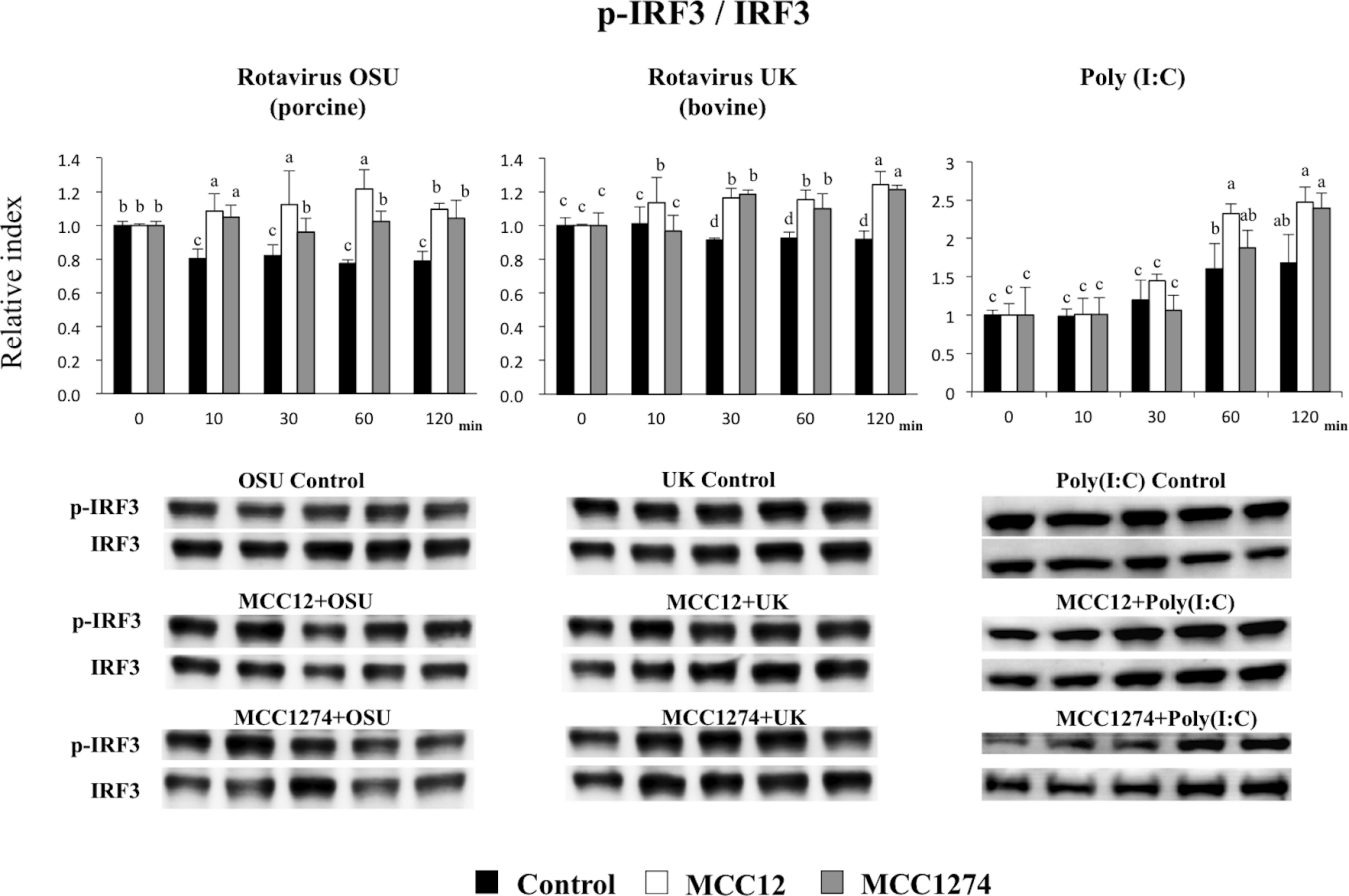
Effect of immunobiotic bifidobacteria on IRF3 activation in porcine intestinal epithelial cell line (PIE cells). PIE cells were pre-treated with *B*. *infantis* MCC12 or *B*. *breve* MCC1274, and then challenged with rotaviruses OSU or UK; or poly(I:C). Total proteins were extracted from lysed cells and separated by SDS-PAGE, and then western-blot was performed to analyze phosphorylation of IRF3 after 10, 30, 60 and 120 minutes. The phosphorylated IRF3 specific bands were normalized to that corresponding to total IRF3. Intensities of proteins bands were calculated from peak area of densitogram by using image software. The results represent data from three independent experiments and are expressed as relative index vs 0 min control with mean ± S.D. ^a, b, c^ Different superscripts letters indicate significant difference (p<0.05) among stimulants at the same time point.

Finally, we aimed to evaluate whether bifidobacteria were able to modulate expression of TNF-receptor associated factor 3 (TRAF3) and interferon regulatory factor 3 (IRF3) in response to OSU and UK RVs inoculation and poly(I:C) treatment. Both bifidobacteria strains were capable of increasing TRAF3 expression in poly(I:C)-challenged PIE cells, with *B*. *breve* MCC1274 being more efficient than *B*. *infantis* MCC12 (Fig 8). In addition, TRAF3 was significantly increased in MCC1274- and MCC12-treated PIE cells after the infection with OSU or UK RVs (Fig 8). Increased expression of IRF3 was also observed in *B*. *infantis* MCC12- and *B*. *breve* MCC1274-treated PIE cells after poly(I:C), OSU or UK challenges (Fig 9).

## Discussion

Rotavirus is a major cause of acute and severe gastroenteritis in animals and humans. Young animals are more frequently infected by RVs, and the age progresses reduces this susceptibility to RVs infection, which is probably due to the maturation of the immune system, and/or the acquisition of specific immunity [21]. Owing to the close relationship of swine and cattle with humans and the genetic diversity of RVs; porcine and bovine RVs are considered a large potential genetic pool for human RVs. In addition, these RVs are important livestock pathogens that reduce growth performance, increase the mortality of animals, and have a deep economic impact worldwide. Therefore, better understanding of the molecular mechanisms that are involved in the defense against RVs are indispensable for the development of new prevention strategies to protect or treat animals and humans [22].

Few studies investigated the infection and immune responses of RVs by using bovine or porcine IECs. The porcine small intestinal epithelial (IPEC-J2) cell line was used by Liu et al. [12] to evaluate RVs infection. The study demonstrated that the porcine RVs strain OSU is able to induce a more severe cytopathic effect and reduced viability of IPEC-J2 cells when compared to human Wa RVs. Similarly, we demonstrated here that PIE cells were infected with porcine OSU or bovine UK RVs, but not infected with human Wa and murine EW RVs.

**Fig 10:**
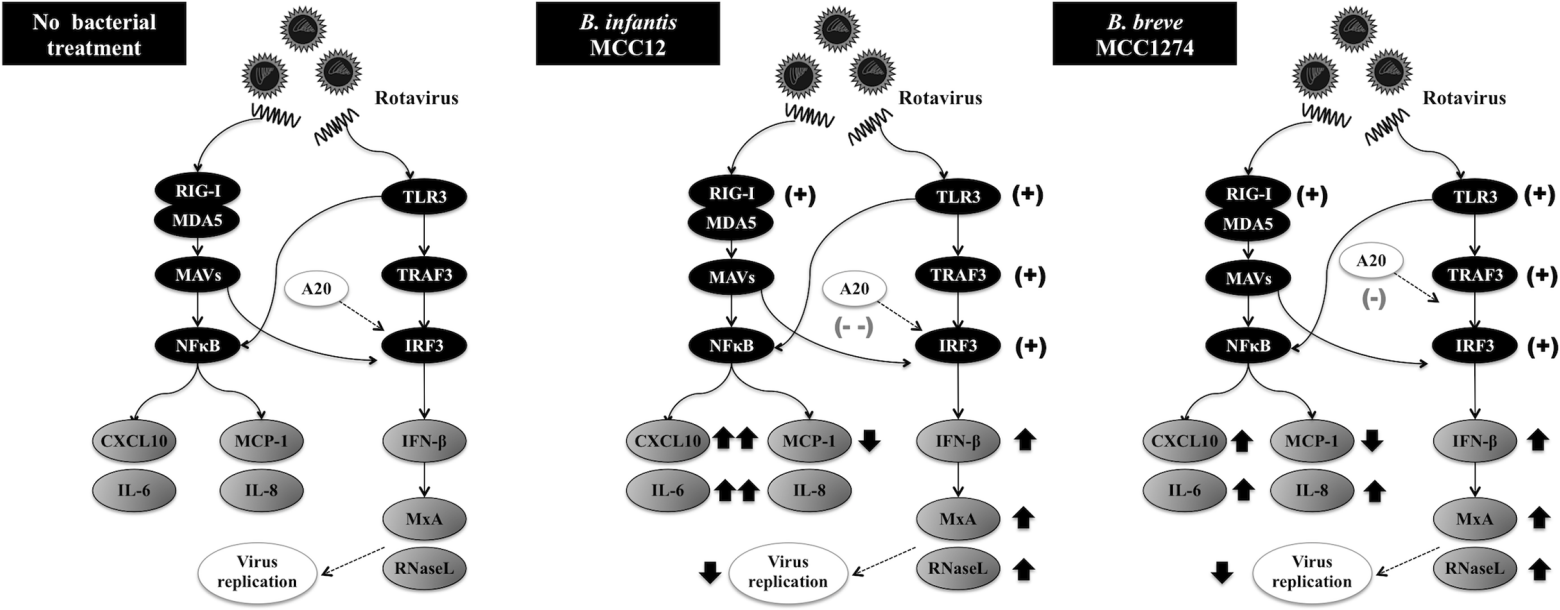
Proposed mechanism. The possible immunomodulatory activity of *B*. *infantis* MCC12, and *B*. *breve* MCC1274 in porcine intestinal epithelial cell line (PIE cells) after stimulation with rotaviruses. Arrows indicate up and down-regulation of cytokines/chemokines, and anti-viral factors. (+): upregulation, (--): strong down-regulation, (-): moderate down-regulation.

We also demonstrated here that PIE cells are able to mount antiviral responses against RVs. A critical and virtually universal early innate response of the host cell to viral infection is the secretion of cytokines belonging to the IFN family. The secretion of IFN results in the expression of several ISG products with antiviral activities. RVs infection stimulates IFN-β and early antiviral gene expression by a signaling pathway that requires MAVS, an adaptor protein that is recruited to signaling complexes following activation of RIG-I or MDA-5 [23, 24]. Interestingly, both RIG-I and MDA-5 are involved in recognizing RVs infection and loss of either of these factors substantially decreases the magnitude of IFN-β induction [23, 24]. In addition, viral RNA can be detected in the host cell by TLR3, which signals through the TIR-domain-containing adaptor-inducing IFN-β (TRIF). RVs replication is then recognized by TLR3, RIG-I and/or MDA-5, which in turn active IRF3 and NF-κB and induce expression of ISGs. Among the ISGs, MxA and RNase L have important roles in antiviral defenses. MxA is an IFN-induced dynamin like GTPase, which provides immune defense against most viral infections including DNA and RNA viruses by exerting antiviral effect [25]. MxA is capable to bind with nucleoproteins of virus and reduce viral replication, although the exact mechanism is still unclear [26]. On the other hand, RNase L is a 2’-5’ oligoadenuylate dependent endoribonulecase that play a pivotal role in the anti-proliferative and anti-viral activity of IFNs [27]. Type I IFNs induce activation of RNase L by interacting with 2-5A oligoadinylate synthetases, which results in cleavage of viral RNA. Our results showed that PIE cells are able to activate TLR3-, RIG-I- and MDA-5-mediated immune responses with activation of IRF3 and NF-κB, induction of IFN-β and up-regulation of the ISGs MxA and RNase L (Fig 10). Then, considering that PIE cells are permissive to porcine RVs and mount classical innate antiviral responses, this cell line could be a useful *in vitro* tool for the study of porcine RVs infection, pathogenesis, and molecular innate immune responses as well as for the evaluation of treatments aimed to improve antiviral defenses and/or modulate inflammatory-mediated damage.

Accumulating evidences suggest that orally administrated probiotics could play a role in maintaining intestinal immune system and increasing the defense against invading pathogenic microbes including viruses. Lactobacilli are commensals found in human and porcine intestinal tracts and are also one of the major gram-positive microorganisms used as probiotics. Among lactobacilli, several beneficial effects against RVs have been documented for the probiotic strain *Lactobacillus rhamnosus* GG. These include shortening the duration of RVs diarrhea, reducing the number of diarrhea episodes, lessening RVs shedding, normalizing gut permeability, and increasing the production of RVs-specific antibodies [28–30]. Some studies have also documented antiviral capacities of the GG strain in porcine systems, although the mechanisms behind this beneficial effect are not well understood.

It was reported that GG strain had a clear and moderate protective effect on RVs-induced diarrhea and virus replication in gnotobiotic pigs [13]. During RVs infections, disruption of the barrier function in IECs cultures *in vitro* and development of villous atrophy *in vivo* have been observed. Those effects have been partially associated to alterations of TGF-β production and intestinal epithelial tight junctions barrier alterations. It was showed that treatment of pigs with *L*. *rhamnosus* GG attenuated the decrease of TGF-β and promoted the enhancement of intestinal epithelial tight junctions, which may contribute to the preservation and restoration of the gut homeostasis after RVs infection [13]. By using the IPEC-J2 cell line, it was also reported that *L*. *rhamnosus* GG is able reduce mucin secretion response induced by porcine RVs, and down-regulated IL-6 which would reduce inflammatory damage [12]. To our knowledge, no detailed molecular mechanisms of anti-RVs probiotic actions have described in porcine systems. Moreover, few studies about the potential beneficial effects of bifidobacteria against RVs have been reported in pigs. *In vivo* studies showed that administration of bifidobacteria were able to colonize the intestine of gnotobiotic pigs and to reduce RVs shedding titers and diarrhea [7, 31].

Previously, we used PIE cells for the selection of LAB strains with the capacity to modulate poly(I:C)-triggered immune response considering the enhancement of IFN-β production [14, 15]. We used the same approach here to select potential bacteria with anti-RVs effect. Of the strains evaluated in our study, *B*. *infantis* MCC12 and *B*. *breve* MCC1274 significantly up-regulated IFN-β in response to poly(I:C) challenge in PIE cells, therefore both strains were selected to further antiviral studies. Our results showed that PIE cells treated with MCC12 and MCC1274 strains significantly increased their resistance to RVs infection, while other strains with moderate or no effect in IFN-β production did not affected RVs replication (data not shown). This is in line with the observation that IFN-β is a key factor for improving defenses against RVs since their replication is restricted by pretreatment of permissive cells with IFN-β [22]. Likewise, treatment of newborn calves and piglets with IFN-β prior to RVs infection suppresses virus replication and reduces disease severity (reviewed in [32]). In addition, *B*. *infantis* MCC12 and *B*. *breve* MCC1274 significantly up-regulated the expression of ISGs MxA and RNase L probably related to the higher levels of IFN-β. RNA fragments produced by RNase L reduce viral replication by activating RLRs to amplify IFN production and by inducing apoptosis to eliminate infected cells [33]. On the other hand, Mx family members have distinct antiviral profiles against a diverse range of viruses, among them pathogens of great importance in human and veterinary medicine [34]. MxA inhibits viruses by sequestering the newly synthesized viral proteins into perinuclear complexes. Then, the up-regulation of MxA, RNase L and probably other ISGs induced by MCC12 and MCC1274 strain through IFN-β would be related to the lower RVs replication found in bifidobacteria-treated PIE cells (Fig 10).

In addition to IFN, the induction of numerous cytokines is an important part of the innate response in virus-infected cells. Initial studies of cytokine induction by RVs utilized IEC line infection models. In HT-29 cells a robust induction of the chemokine IL-8 was observed following RVs infection [35]. The induction of IL-8 by RVs was found to be caused by NF-κB activation and binding to the IL-8 promoter [36]. In addition to IL-8, several other cytokines and chemokines that at least partially rely on NF-κB for their induction have been detected in IEC lines and mouse intestines following RVs infection. These include RANTES, GM-CSF, GRO-α, MIP-1β, and IP-10 [37]. Although this mechanism represents an important primary line of host defense, prolonged or dysregulated pro-inflammatory cytokine production may lead to tissue damage and epithelial barrier dysfunction. In this regard, we have previously reported that regulation of inflammatory response induced by probiotic bacteria is essential to achieve full protection against pathogens [38, 39]. Results of the present work are in line with our previous data since *B*. *infantis* MCC12 and *B*. *breve* MCC1274 significantly reduced the production of IL-8 in RVs-infected PIE cells.

TLR negative regulators play key roles in maintaining intestinal hemostasis by regulating TLR signaling. The zinc-finger protein A20, due to its deubiquitinase and ubiquitinase E3 ligase activities, is capable to terminate TLR signaling which results in inhibition of NF-κB activation and reduction of inflammatory induced cytotoxicity [40]. Saitoh et al. [41] reported that IRF-3 activation is suppressed by A20. The A20 is able to induce the suppression of the IFN stimulation response element- and IFN-promoter-dependent transcription by physically interacting with NF-κB-activating kinase/Traf family member-associated NF-κB activator-binding kinase 1 and IKK-i/IKKε; and inhibiting dimerization of IRF-3 following engagement of TLR3 by dsRNA or Newcastle virus infection. Importantly, knocking down of A20 expression results in enhanced IRF-3-dependent transcription triggered by the stimulation of TLR3 or virus infection. It was also demonstrated that human monocyte-derived dendritic cells (DCs) activated with poly(I:C) up-regulate A20 and that down-regulating this protein has an important role in the functional activation of DCs. A20 down-regulated DCs showed higher activation of the transcription factors NF-κB and AP-1, which resulted in increased and sustained production of IL-6, IL-10, and IL-12p70, and DCs with enhanced T cell stimulatory capacity [42]. We observed that both *B*. *infantis* MCC12 and *B*. *breve* MCC1274 significantly reduced the expression of A20 in RVs-infected PIE cells, which is in line with the capacity of both strains to improve IRF-3 activation and IFN-β production (Fig 10). Similarly, MacPherson et al. [43] studied the effect of probiotics in the modulation of poly(I:C) induced inflammatory response in HT-29 cells. Cells were challenged with poly(I:C) in the presence or absence of probiotic strains. Stimulation with the TLR3 agonist alone increased the expression of A20, however significantly reduced levels of expression were observed in cells co-challenged with poly(I:C) and probiotics. The receptor(s) that are activated by MCC12 and MCC1274 in PIE cells to reduce A20, improve IRF-3 activation and increase IFN-β production remains to be uncovered. We demonstrated previously that bifidobacteria strains with a high capacity to stimulate TLR2 such as *B*. *longum* BB536 and *B*. *breve* M-16V were able to increase the expression of A20 in PIE cells and reduce TLR4-mediated inflammatory response [19, 44]. On the contrary, strains with low capacity of stimulating TLR2 did not modify the expression of the ubiquitin-editing enzyme A20 in PIE cells challenged with TLR4 agonists. In addition, we recently showed that the probiotics strains *L*. *rhamnosus* CRL1505 and *L*. *plantarum* CRL1506 are able to improve the production of type I IFNs in PIE cells and that anti-TLR2 or anti-TLR9 antibodies were not able to block the increase of IFN-β induced by the lactobacilli [15]. Then, TLR2 or TLR9 would not be involved in the antiviral capabilities of *B*. *infantis* MCC12 and *B*. *breve* MCC1274. Further studies are needed in order to find the PRRs involved in the recognition of bifidobacteria leading to A20 and IFN-β modulation in PIE cells.

## Concluding Remarks

RVs are considered major gastroenteritis pathogens in swine because they are responsible for significant economic losses due to its increased mortality, treatment costs, and reduced weight gain [22]. The study of innate immune responses to RVs in the porcine host is fundamental to improve prevention and treatment strategies. We demonstrated here that porcine and bovine RVs are able to infect PIE cells, which then mount classical innate antiviral responses including TLR3-, RIG-I- and MDA-5-mediated immune responses with activation of IRF3 and NF-κB, induction of IFN-β and up-regulation of ISGs. These results indicate the value of this porcine cell line for studying RVs molecular innate immune response in the pig. In addition, PIE cells can be used for the selection and mechanistic studies of probiotic bacteria with the capacity to improve resistance against RVs. In this regard, we showed that innate immune response against RVs was efficiently modulated by *B*. *infantis* MCC12 and *B*. *breve* MCC1274 in PIE cells. Our results suggest that these two bifidobacteria strains have potential to be used as antiviral substitutes to reduce severity of RVs infection in animals. Moreover, several studies of gastrointestinal physiology as well as mucosal immunobiology have demonstrated that pigs are expected to be a better human model than rodents. Therefore, PIE cell could be also used for the selection and study of antiviral therapies for application in humans.

## Supporting Information

S1 Table Primers used in this study. (DOCX)
