# Supplementary material for: Immunobiotic Bifidobacteria Strains Modulate Rotavirus Immune Response in Porcine Intestinal Epitheliocytes via Pattern Recognition Receptor Signaling
Source: PLoS One. 2016 Mar 29;11(3):e0152416. doi: 10.1371/journal.pone.0152416 (PMC4811565; doi:10.1371/journal.pone.0152416)
Supplement: S1 Table — (DOCX) [file pone.0152416.s001.docx]

Table 1. **Primer sequences used for qRT-PCR in this study**

Sense primer Antisense primer

Porcine β-actin CATCACCATCGGCAACGA GCGTAGAGGTCCTTCCTGATGT

Porcine IL-6 TGGATAAGCTGCAGTCACAG ATTATCCGAATGGCCCTCAG

Porcine IL-8 GCTCTCTGTGAGGCTGCAGTT TTTATGCACTGGCATCGAAGTT

Porcine MCP-1 ACAGAAAGAGTCACCAGCAGCAA GCCCGCGATGGTCTTG

Porcine IFN-β AGTTGCCTGGGACTCCTCAA CCTCAGGGACCTCAAAGTTCAT

Porcine MxA GAGGTGGACCCCGAAGGA CACCAGATCCGGCTTCGT

Porcine RNaseL GCAGCCGAGCCAACGATA AGCTCCCGTCGCTCTCACT

Porcine RIG-I CCCTGGTTTAGGGACGATGA GTCGGGCCCTTGTTGTTTTT

Porcine TLR3 TAGAGACATGGATTGCTCCC AACTTCTGGAATGCAGGTCC

Porcine A20 CCTCCCTGGAAAGCCAGAA GTGCCACAAGCTTCCTCACTT
